# Chemical Burns in Northern China: Co-Occurrence of Low TBSA and Recorded Full-Thickness Involvement in a 7-Year Retrospective Cohort

**DOI:** 10.3390/ebj7030049

**Published:** 2026-09-21

**Authors:** Tian Liu, Hui Zhou, Fangchao Hu, Yirui Qu, Xiangyu Liu, Chengfeng Xu, Yunfei Chi

**Affiliations:** 1 Department of Outpatient, The Fourth Medical Center of Chinese PLA General Hospital, Beijing 100048, China; 2 Senior Department of Hematology, The Fifth Medical Center of Chinese PLA General Hospital, Beijing 100071, China; 3 Graduate School, Chinese PLA General Hospital, Beijing 100853, China; 4 Department of General Surgery, The Ninth Medical Center of Chinese PLA General Hospital, Beijing 100101, China

**Keywords:** chemical burns, burn epidemiology, occupational injuries, burn depth, resource utilization

## Abstract

**Highlights:**

**What are the main findings?**

- Workplace exposure was recorded in 47.2% of the 212 included patients with chemical burns;
- Among patients with ≤2% TBSA, 87 of 105 patients with a known depth had a recorded full-thickness component, and 81 of 109 had a positive recorded III/IV-degree area.

**What are the implications of the main findings?**

- Within this selected inpatient cohort, low TBSA frequently co-occurred with a recorded full-thickness component. This observation does not establish a triage threshold.
- Occupational injury prevention and standardized prospective documentation are priorities.

**Abstract:**

**Background:** Total body surface area (TBSA) quantifies burn extent, whereas burn depth reflects tissue involvement. In chemical burns, small surface-area injuries may still include deeper tissue damage, but this patient-level co-occurrence remains incompletely described; we therefore examined it in hospitalized patients in northern China. **Methods:** We retrospectively analyzed a merged 2016–2022 burn database. Records coded as chemical burns were included when the recorded length of stay exceeded one day. **Results:** Of 5929 screened records, 214 were coded as chemical burns. Exclusion of two short-stay records left 212 unique patients. Most were male (74.5%) and aged 18–60 years (84.9%). Workplace exposure was recorded for 47.2%. Among 105 patients with ≤2% TBSA and a known depth, 87 (82.9%; 95% confidence interval [CI]: 74.5–88.9%) had a recorded maximum-depth category involving a full-thickness component. Among all 109 patients with ≤2% TBSA, 81 (74.3%; 95% CI: 65.4–81.6%) had a positive recorded III/IV-degree area. Acids and alkalis accounted for 30.2% and 29.7% of recorded exposures, respectively, and 37.3% underwent at least one operation. **Conclusions:** Working-age adults predominated, and workplace exposure was the most common recorded setting. Low TBSA frequently co-occurred with recorded full-thickness involvement in this selected inpatient cohort. The findings support reporting recorded burn depth alongside TBSA in hospitalized chemical injuries and improving standardized prospective documentation.

## 1. Introduction

Burn injuries cause substantial morbidity, mortality, and long-term functional impairment [1]. Chemical injuries may continue to progress after exposure and may also cause agent-specific systemic toxicity [2,3]. Burn depth is commonly described as superficial, superficial dermal, deep dermal, or full-thickness. Superficial dermal and deep dermal injuries correspond to superficial and deep partial-thickness injuries, respectively [4]. Chemical agents include acids, alkalis, and heterogeneous reactive substances or preparations. Acids commonly cause coagulative tissue injury, whereas alkalis can cause liquefactive injury and continued penetration. These are general patterns, and hydrofluoric acid has distinctive local and systemic toxicity [3,5].

Chemical-burn patterns vary with industrial activity, workplace practice, safety regulations, and household chemical use. Published series show wide variation in the proportion of burn admissions caused by chemicals and in the causative agents, severity, and treatment requirements [6,7,8,9]. A multicenter study in mainland China found that chemical injuries accounted for 3.47% of burns [10]. Studies from southwestern China and South Korea provide additional information on exposure patterns and injury characteristics [11,12]. These differences make regional data important for prevention and clinical preparedness.

The percentage of total body surface area (TBSA) burned quantifies burn extent, whereas depth describes tissue involvement. Regional studies from Guangdong, southwestern China, western Zhejiang, and Beijing have characterized chemical exposures, burn size, depth, and treatment requirements [7,11,13,14]. Joint patient-level reporting of burn area and depth can further clarify injury patterns among patients with small burns. Accordingly, we examined the maximum-depth category and separately recorded III/IV-degree area within the lowest TBSA category, retaining records with missing or discordant depth/area fields and assessing the influence of short hospital stays and hydrofluoric acid exposures.

In this study, we analyzed a 7-year cohort from a tertiary burn center in northern China. The primary objective was to characterize demographic and recorded exposure patterns, injury severity, and the patient-level co-occurrence of TBSA category, maximum-depth category derived from source codes, and recorded III/IV-degree burn area. Secondary exploratory objectives were to describe recorded in-hospital management, microbiological documentation, antibacterial-agent selections, resource utilization, and administrative care disposition.

## 2. Materials and Methods

### 2.1. Study Design and Participants

This retrospective single-center observational study included patients admitted to a tertiary burn center in northern China from 1 January 2016 through to 31 December 2022. Reporting followed the Strengthening the Reporting of Observational Studies in Epidemiology statement [15].

All 5929 records in the merged burn database were screened. Records were eligible when the cause-of-injury field identified a chemical burn and the recorded length of stay exceeded one day. Two records with a recorded stay of one day were excluded; this criterion defined the selected inpatient cohort. Records generated by administrative re-registration within the same clinical hospitalization were consolidated during database preparation. Unique patient identifiers were checked, and changes in registration number within the same clinical hospitalization were not counted as separate readmissions. No formal sample-size calculation was performed because all eligible records during the study period were included.

### 2.2. Ethics Statement

The study was approved by the Ethics Committee of the Fourth Medical Center of Chinese PLA General Hospital (Approval No.: 2021KY033-KS001). The requirement for informed consent was waived by the ethics committee because of the retrospective design and use of de-identified clinical data. The study was conducted in accordance with the Declaration of Helsinki and applicable institutional requirements.

### 2.3. Data Sources and Variable Definitions

Data were extracted by one researcher and checked by a second researcher using the database fields and coding guide.

Demographic variables included age and sex. Age was calculated from the date of birth and admission date and categorized as ≤17 years, 18–40 years, 41–60 years, and ≥61 years. Injury settings were classified as workplace, household, public building, outdoor environment, or other/uncertain based on available records.

Recorded exposures were classified using the cause-of-injury descriptions and the source coding guide. Named acids and alkalis were retained within their parent groups, while heterogeneous products or preparations were reported separately. Hydrofluoric acid was counted only when explicitly named. Two ambiguously named fluorinated-acid records were classified under the “other/acidic mixture” category. Other or insufficiently specified exposures were retained as a separate category (File S1).

Burn severity was described using recorded TBSA, maximum-depth categories, and the separately recorded III/IV-degree area. TBSA categories were ≤2%, >2–5%, >5–10%, >10–30%, and >30%. Source Code 1 comprised superficial and superficial dermal injuries. The three Code 1 records in this cohort described superficial dermal injury. Code 3 denoted deep dermal injury. Code 2 and text-only entries “2” or “s2-d2” were classified as mixed or unspecified dermal injuries. Codes 4–7 denoted injury with a full-thickness component, including mixed dermal/full-thickness injury and injury extending beyond skin. This grouping did not imply that the entire burn area was full thickness. Missing or unclassifiable depth records were categorized as uncertain. The source database separately recorded III/IV-degree burn area using its original grading terminology. This terminology was retained when referring specifically to that source field. Recorded III/IV-degree area was grouped as 0%, >0–2%, >2–5%, >5–10%, >10–30%, >30%, or uncertain. Recorded depth was based on the treating physicians’ clinical assessments and analyzed as the maximum documented category during hospitalization. Information on standardized assessment timing, assessor identity, and patient-specific use of adjunctive instruments was unavailable. The TBSA, depth, and area fields were cross-tabulated at the patient level without independent adjudication against operative findings (Table S1).

Admission and discharge dates were available for all patients. Injury dates were considered complete only when a valid calendar date was recorded; partial, relative, malformed, or missing dates were not imputed.

### 2.4. Clinical Management and Microbiological Variables

Recorded management variables included the number of operations, cumulative operating time, microbiological records, and antibacterial-agent selections. Surgical procedure types, comprehensive data on non-antibacterial inpatient medications, agent-specific antidotal treatments, and information on alcohol or drug intoxication contributing to the injury were not systematically available.

Microbiological variables were extracted from recorded bacterial categories and identified bacterial taxa. Because the database did not distinguish between absence of specimen collection and negative culture results, microbiological findings were interpreted descriptively.

Recorded antibacterial-agent selections were summarized according to the documented agents. Standardized information on indication, sampling context, administration timing, route, duration, and susceptibility was unavailable for these recorded antibacterial-agent selections. They were therefore not used to diagnose infection or evaluate prescribing appropriateness.

### 2.5. Care Disposition and Resource Utilization

Care disposition was obtained from the administrative database and classified as “healed or no further wound care recorded,” “outpatient wound care,” “readmission,” or “discharge in critical condition.”

Because care disposition was an administrative field without a standardized follow-up period, the recorded readmission category had no uniform time window, and disposition was not interpreted as a long-term clinical outcome.

Length of stay was calculated from admission to discharge. Hospital costs were analyzed in nominal Chinese yuan (RMB) without adjustment for inflation or changes in hospital pricing. No missing-value imputation was performed. Missing or uncertain values were reported explicitly, and each analysis used the available-case denominator for the variables involved.

### 2.6. Statistical Analysis

Categorical variables are presented as counts and percentages. Continuous variables are presented as medians with first and third quartiles because normality was not assumed. Categorical comparisons were performed using Fisher–Freeman–Halton exact tests.

Continuous variables were compared using Kruskal–Wallis tests for more than two groups and Wilcoxon’s rank-sum tests for two groups. Wilson score 95% confidence intervals (CIs) were calculated for the low-TBSA proportions because they provide bounded binomial intervals with more reliable coverage than simple Wald intervals [16]. Hodges–Lehmann location-shift estimates and rank-based 95% CIs were used to summarize the unadjusted depth comparisons for skewed resource-use data [17]. These estimates represent the median of between-group pairwise differences, rather than the difference between sample medians. A common location-shift interpretation assumes similarly shaped distributions. All tests were two-sided.

To address confounding by burn area, exploratory ordinary least-squares linear models were fitted separately to the natural logarithms of length of stay and hospital cost. Each model included an indicator for a recorded full-thickness component, a natural cubic spline of log(1 + TBSA) with three degrees of freedom, age per 10 years, sex, and admission year centered at 2019. These covariates were included to account for burn extent and demographic and temporal differences. Complete cases were used. Exponentiated depth coefficients were reported as adjusted geometric-mean ratios, with heteroskedasticity-consistent type 3 (HC3) robust standard errors and residual-degree-of-freedom t intervals [18]. Model rank, residuals, leverage, and influence were examined. Additional model specifications and diagnostics are provided in the Tables S2 and S3.

Sensitivity analyses repeated the two focal ≤2% TBSA proportions after including the two excluded one-day records and after excluding explicitly recorded hydrofluoric acid exposures. A broader exclusion also removed the two ambiguously named fluorinated-acid records (Table S4). These sensitivity analyses and adjusted models were exploratory, without multiplicity correction or data-driven variable selection. Analyses were performed using R version 4.4.3 and Python version 3.12.4.

## 3. Results

### 3.1. Cohort Identification and Demographic Characteristics

Of 5929 screened database records, 214 (3.61%) were coded as chemical burns. Excluding the two records with a recorded stay of one day left 212 unique patients. Recorded admission dates ranged from 5 January 2016 to 3 November 2022, within the study period of 1 January 2016 through to 31 December 2022. A complete, valid injury date was available for 182 patients (85.8%); among these patients, recorded injury dates ranged from 5 January 2016 to 3 November 2022. Twenty-five date entries were partial, relative, or malformed, and five were missing.

**Figure 1:**
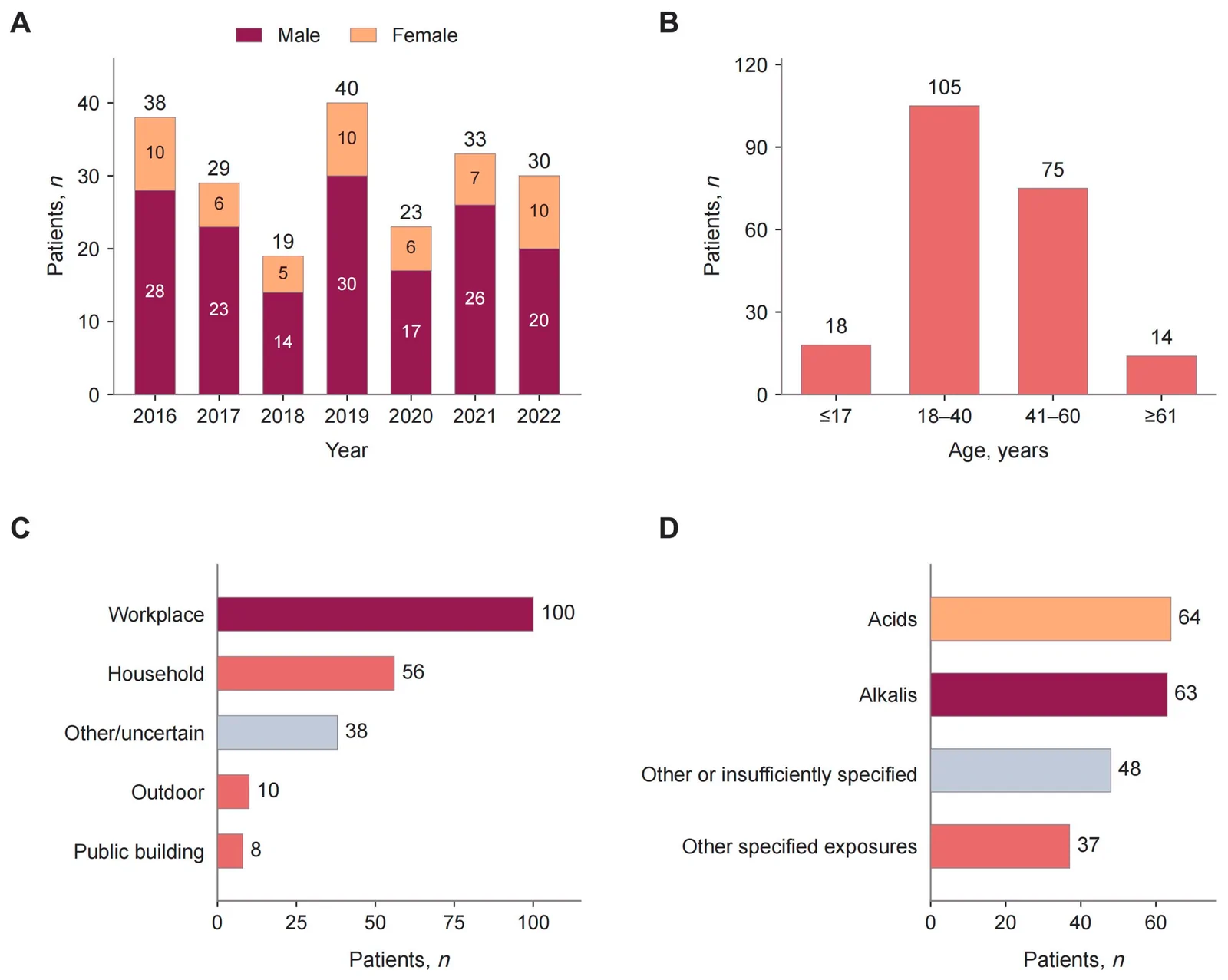
Demographic, epidemiologic, and recorded exposure characteristics of the 212 included patients: (**A**) Annual patient counts by sex from 2016 through 2022. (**B**) Age distribution. (**C**) Injury setting. (**D**) Recorded exposure groups. “Other specified exposures” comprises tar or bitumen, cement, pesticide, garlic, herbal preparation, and antifreeze.

The cohort included 158 male patients (74.5%) and 54 female patients (25.5%). Most patients were working-age adults, with 180 patients (84.9%) aged 18–60 years. Annual numbers of patients with chemical burns ranged from 19 to 40 during the study period (Figure 1).

Workplace exposure was the most common injury setting, occurring in 100 patients (47.2%), followed by household exposure in 56 patients (26.4%). Other settings included public buildings, outdoor environments, and unspecified or other circumstances (Figure 1).

Among the 56 household exposures, recorded products included drain-cleaning products (*n* = 8), adhesives (*n* = 4), topical herbal preparations (*n* = 4), and garlic preparations (*n* = 3). Workplace safety practices and protective equipment were not systematically documented.

### 3.2. Exposure Patterns and Injury Characteristics

**Figure 2:**
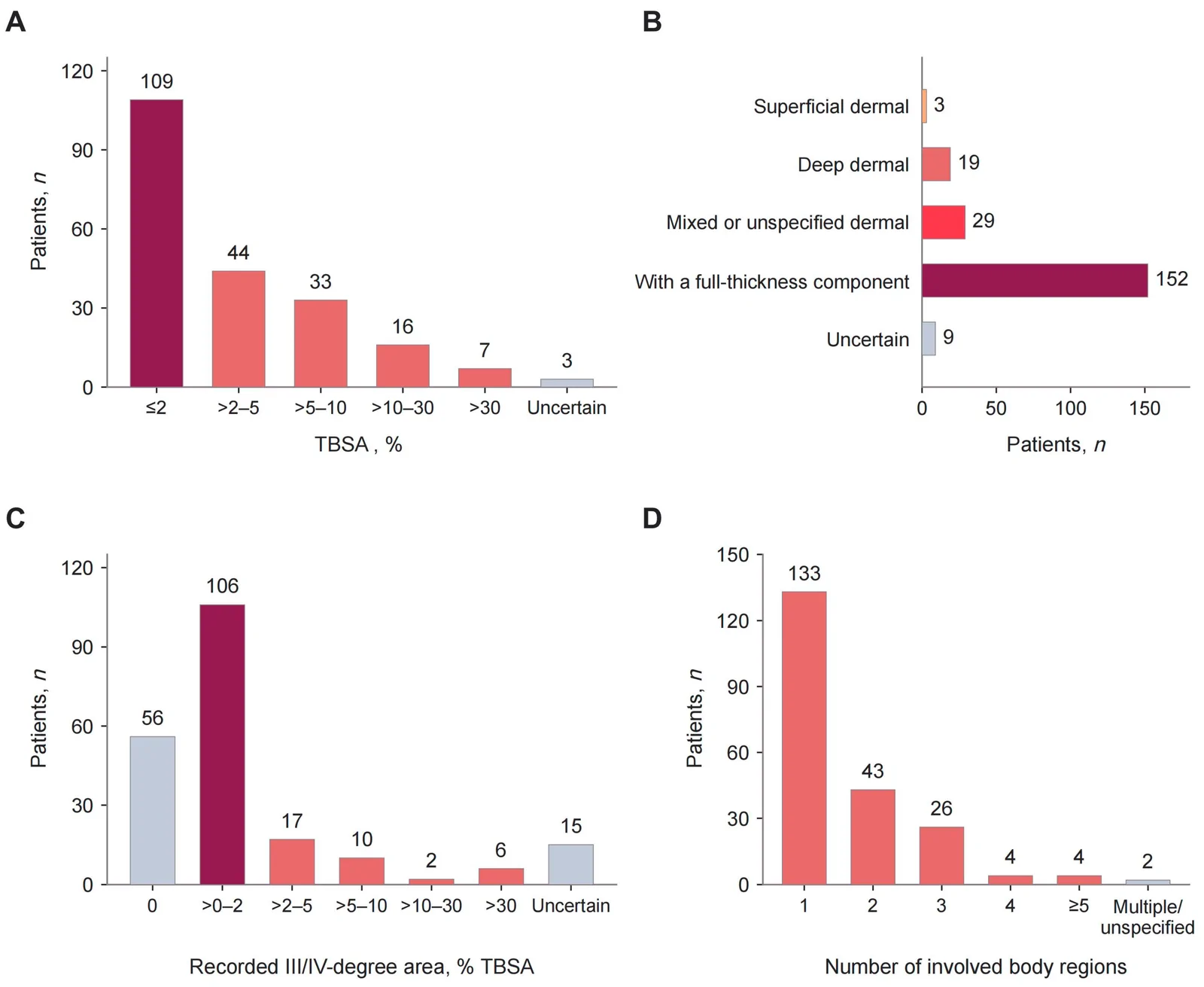
Burn size and recorded injury characteristics of 212 patients: (**A**) Total body surface area (TBSA) category. (**B**) Recorded maximum-depth category; mixed or unspecified dermal entries are displayed separately from explicitly deep dermal entries. (**C**) Recorded III/IV-degree area. (**D**) Number of involved body regions. Uncertain categories are retained. Codes 4–7 indicate a full-thickness component, potentially extending beyond skin, and do not specify that the entire burn area was full thickness.

The extremities were involved in 110 patients (51.9%); the head, face, or neck in 65 patients (30.7%); and the hands in 48 patients (22.6%) (Table 1). One body region was recorded for 133 patients (62.7%). The remaining patients had multiple or unspecified region counts (Figure 2D).

TBSA was known for 209 patients, and 109 of the 212 patients (51.4%) were in the ≤2% TBSA category. Among the 105 patients in this subgroup with a known depth, 87 (82.9%; 95% CI: 74.5–88.9%) had a recorded full-thickness component. A positive III/IV-degree area was recorded in 81 of the 109 patients (74.3%; 95% CI: 65.4–81.6%), or 81 of the 103 with a known area (78.6%; 95% CI: 69.8–85.5%). The joint distribution included zero and uncertain area records. Across the cohort, 152 patients (71.7%) had a maximum-depth category involving a full-thickness component. The complete depth distribution is shown in Table 1 and Figure 2.

Acids and alkalis accounted for 64 (30.2%) and 63 (29.7%) recorded exposures, respectively. Hydrofluoric acid was explicitly named in 23 records (10.8%), all within the ≤2% TBSA group; two other fluorinated-acid descriptions were insufficiently specific to identify hydrofluoric acid. Sodium hydroxide was the most frequently named alkali (23 patients, 10.8%). Other or insufficiently specified exposures accounted for 48 patients (22.6%) (Figure 1D and Table 2).

Table 3 shows the joint distribution of recorded maximum-depth category and III/IV-degree area within the ≤2% TBSA subgroup, including zero and uncertain area records.

### 3.3. Clinical Management and Microbiological Documentation

**Figure 3:**
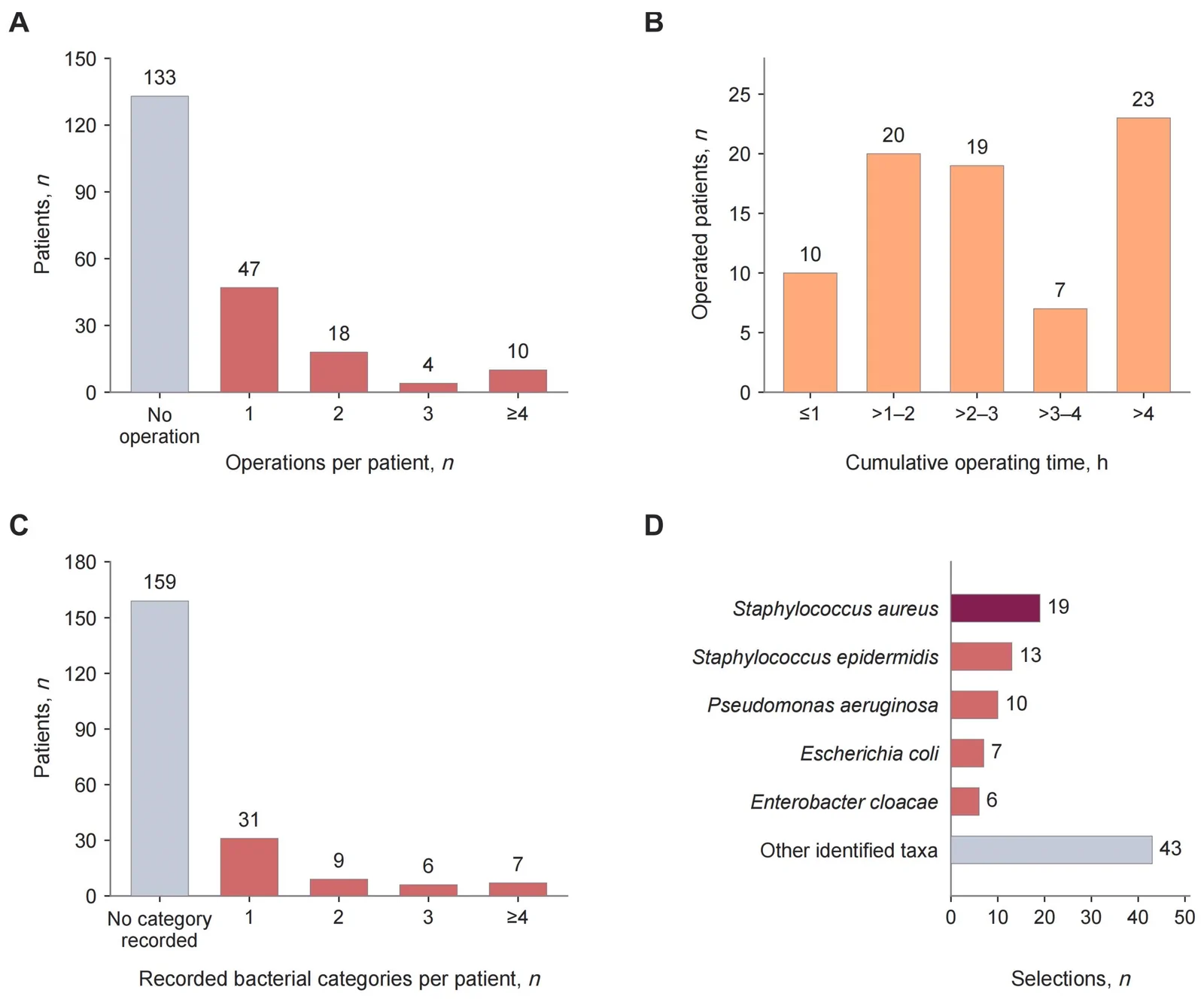
Clinical management and microbiological documentation: (**A**) Operations per patient (*N* = 212). (**B**) Cumulative operating time among 79 operated patients. (**C**) Number of bacterial categories recorded per patient (*N* = 212). The absence of a recorded category does not distinguish lack of sampling from a negative result. (**D**) Ninety-eight selections with an identified bacterial taxon are shown; 18 unspecified bacterial selections were not plotted. Patients could contribute to more than one selection.

Seventy-nine patients (37.3%) underwent at least one operation. Of these, 47 underwent one operation, 18 underwent two operations, four underwent three operations, and 10 underwent four operations or more. Cumulative operating time exceeded 4 h in 23 of the 79 operated patients (Figure 3).

The available records did not permit classification of operations as excision, debridement, grafting, or other procedures.

At least one bacterial category was recorded for 53 patients (25.0%), and no category was recorded for 159 (75.0%). The database contained 98 selections with an identified bacterial taxon and 18 additional unspecified bacterial selections. Among identified taxa, *Staphylococcus aureus* was the most frequently recorded (19 selections), followed by *Staphylococcus epidermidis* (13) and *Pseudomonas aeruginosa* (10) (Figure 3).

At least one antibacterial agent was recorded for 170 patients (80.2%). Among the 233 recorded antibacterial-agent selections, moxifloxacin was the most frequent specific agent (60 selections), followed by cefmetazole (42) and latamoxef (22) (Figure S1).

### 3.4. In-Hospital Care Disposition and Resource Utilization

Care disposition data were available for 210 patients. Among these patients, 104 (49.5%) were classified as “healed or no further wound care recorded”; 94 (44.8%) as “outpatient wound care”; 11 (5.2%) as “readmission”; and one (0.5%) as “discharge in critical condition.” Disposition was missing for two patients. No death was recorded in the disposition field; post-discharge mortality was unavailable.

Administrative care disposition differed across age groups (*p* = 0.012) and TBSA categories (*p* = 0.006), but no statistically significant differences were detected by sex (*p* = 0.275) or by the presence of a maximum-depth category involving a full-thickness component (*p* = 0.574). Because these analyses were unadjusted, exploratory, and based on an administrative field without a standardized follow-up interval, they should be interpreted cautiously (Table 4).

Hospital length of stay and nominal hospital cost differed across TBSA categories (both *p* < 0.001). Patients with a recorded full-thickness component had a higher median length of stay than those without a recorded component (15.5 [7.0–31.0] vs. 10.0 [7.5–15.0] days; *p* = 0.013). The Hodges–Lehmann location-shift estimate was 4.0 days (95% CI: 1.0–9.0). The corresponding median nominal hospital costs were RMB 15,305 [6842–36,698] and RMB 10,537 [6024–15,752] (*p* = 0.016), with a Hodges–Lehmann location-shift estimate of RMB 4080 (95% CI: 741–9295) (Table 5). These unadjusted comparisons describe group differences and do not estimate an independent effect of burn depth.

The adjusted models included 202 patients, of whom 151 had a recorded full-thickness component and 51 did not. Ten patients were excluded because TBSA or depth status was missing or uncertain. For patients with versus without a recorded full-thickness component, the adjusted geometric-mean ratio was 1.57 for length of stay (95% CI: 1.24–1.99; *p* < 0.001) and 1.91 for nominal cost (95% CI: 1.50–2.44; *p* < 0.001). Additional model specifications are reported in Table S2.

Within the ≤2% TBSA subgroup, including the two excluded short-stay records yielded proportions of 82.2% with a recorded full-thickness component and 73.9% with a positive recorded III/IV-degree area. Excluding explicitly recorded hydrofluoric acid exposures changed these proportions to 78.3% and 69.8%, respectively. The broader exclusion of both explicit and ambiguously named fluorinated-acid records yielded corresponding proportions of 79.0% and 70.2%. Counts, denominators, and 95% CIs are provided in Table S4.

## 4. Discussion

Of 5929 burn-database records screened over 7 years, 214 were coded as chemical burns and 212 met the inpatient inclusion criteria. Patients were predominantly working-age adults, and workplace exposure was the leading setting. Within the lowest TBSA category, 82.9% of patients with a known depth had a maximum-depth category involving a full-thickness component, and 74.3% of all patients in that category had a positive recorded III/IV-degree area. Acids and alkalis were the leading recorded exposure groups, and exploratory analyses further characterized in-hospital management and resource use.

TBSA and burn depth capture different dimensions of injury: extent and tissue involvement, respectively. In chemical burns, ongoing tissue reactions and deeper penetration may allow these dimensions to diverge [2,8,19]. The co-occurrence of a low burn area and recorded full-thickness involvement within individual patients was therefore the central finding of this study. The maximum-depth category and the separate area field showed the same general pattern. Within the ≤2% TBSA subgroup, four records had a maximum-depth category involving a full-thickness component but a recorded III/IV-degree area of 0%, and two had an uncertain area. Differences in assessment timing, documentation, or coding may contribute to this discordance. Reporting both fields clarifies the pattern without treating either as an independently validated measure or implying that the entire burn area was full thickness.

Regional and international reports provide important context but differ in referral pathways, exposure profiles, and documentation [6,7,9,10,11,12,13,14,20,21]. This cohort adds a patient-level cross-tabulation of recorded III/IV-degree area and maximum-depth category within the low-TBSA subgroup. Comparisons of proportions across centers should account for differences in denominators and admission criteria.

Workplace exposure accounted for nearly half of the cohort, consistent with occupational patterns in earlier reports [14,22]. Household records also identified drain-cleaning products, adhesives, topical herbal preparations, and garlic preparations. These findings can help identify priorities for local prevention and education. Standardized prospective recording of product identity and concentration, occupational task, use of personal protective equipment, and first-aid measures would better inform prevention and emergency response. The present data do not evaluate the effectiveness of these measures.

Hydrofluoric acid was explicitly recorded in 23 patients, all with ≤2% TBSA. Its potential for systemic toxicity may increase the likelihood of specialist referral and prolonged observation even when burn size is small [3]. The two focal low-TBSA proportions remained of similar magnitude after exclusion of explicitly recorded hydrofluoric acid cases, although this sensitivity analysis cannot remove referral bias or uncertainty in agent identification. Including the two excluded short-stay records also had little effect on these proportions. These sensitivity analyses do not address the selection of patients who were managed entirely outside the hospital.

A recorded full-thickness component was associated with greater resource use after adjustment for TBSA, age, sex, and year. These exploratory estimates apply only to the included cohort, vary across model specifications, and do not establish a causal effect of burn depth on length of stay or cost.

Recorded antibacterial-agent selections were common, with at least one agent documented for 80.2% of patients. The database did not distinguish treatment from prophylaxis and lacked standardized information on indication, specimen site, culture sampling, timing, route, duration, or susceptibility. These records cannot establish infection, confirm actual administration, or evaluate prescribing appropriateness; they are reported only to describe documentation and to motivate prospective antimicrobial stewardship research using standardized definitions.

This study has several limitations. First, the single-center inpatient setting and exclusion of short stays may underrepresent minor injuries and enrich the cohort for deeper or more complex cases. Second, case identification and exposure classification relied on database entries, and heterogeneous products may include mixed injury mechanisms. Third, clinical depth assessments lacked standardized timing, assessor identification, and independent adjudication. The separate area field represents related documentation rather than independent validation. Fourth, some clinical details were incomplete, including surgical procedure types, non-antibacterial inpatient medications, alcohol or drug intoxication contributing to injury, and long-term outcomes. Care disposition was administrative and lacked a standardized follow-up interval. Fifth, costs were nominal and were not adjusted for inflation or changes in hospital pricing. Adjustment for admission year cannot fully account for changes in clinical care during 2016–2022. Finally, the multivariable models and sensitivity analyses were exploratory and remain subject to residual confounding, documentation error, and model dependence.

Accordingly, the clinical implication is limited to the study population: recording burn depth alongside TBSA provides a more complete description of hospitalized chemical injuries. The ≤2% subgroup does not establish a triage threshold or estimate risk in unselected patients. Existing burn-referral guidance should be considered independently of the proportions reported here [4].

## 5. Conclusions

Within this selected inpatient cohort, low TBSA frequently co-occurred with recorded full-thickness involvement. The findings support reporting recorded burn depth alongside TBSA in hospitalized chemical injuries and improving standardized documentation. Working-age adults predominated, and workplace exposure was the most common recorded setting, highlighting priorities for local prevention. Generalization to unselected patients requires further study.

## Figures and Tables

**Table 1:** Demographic and clinical characteristics of patients with chemical burns stratified by age group.

Characteristic	Total (*N* = 212)	≤17 y (*n* = 18)	18–40 y (*n* = 105)	41–60 y (*n* = 75)	≥61 y (*n* = 14)
Head, face, or neck	65 (30.7)	2 (11.1)	42 (40.0)	20 (26.7)	1 (7.1)
Extremities	110 (51.9)	8 (44.4)	55 (52.4)	38 (50.7)	9 (64.3)
Trunk	46 (21.7)	1 (5.6)	32 (30.5)	12 (16.0)	1 (7.1)
Perineum	10 (4.7)	4 (22.2)	1 (1.0)	4 (5.3)	1 (7.1)
Buttocks	17 (8.0)	5 (27.8)	7 (6.7)	4 (5.3)	1 (7.1)
Hand	48 (22.6)	3 (16.7)	21 (20.0)	20 (26.7)	4 (28.6)
Foot	39 (18.4)	4 (22.2)	22 (21.0)	11 (14.7)	2 (14.3)
Total body surface area: ≤2%	109 (51.4)	9 (50.0)	53 (50.5)	40 (53.3)	7 (50.0)
Total body surface area: >2–5%	44 (20.8)	6 (33.3)	19 (18.1)	15 (20.0)	4 (28.6)
Total body surface area: >5–10%	33 (15.6)	2 (11.1)	19 (18.1)	10 (13.3)	2 (14.3)
Total body surface area: >10–30%	16 (7.5)	1 (5.6)	11 (10.5)	4 (5.3)	0 (0.0)
Total body surface area: >30%	7 (3.3)	0 (0.0)	2 (1.9)	4 (5.3)	1 (7.1)
Total body surface area: uncertain	3 (1.4)	0 (0.0)	1 (1.0)	2 (2.7)	0 (0.0)
Maximum depth: superficial dermal	3 (1.4)	1 (5.6)	1 (1.0)	1 (1.3)	0 (0.0)
Maximum depth: deep dermal	19 (9.0)	2 (11.1)	11 (10.5)	5 (6.7)	1 (7.1)
Maximum depth: mixed or unspecified dermal	29 (13.7)	0 (0.0)	20 (19.0)	6 (8.0)	3 (21.4)
Maximum depth: with a full-thickness component	152 (71.7)	15 (83.3)	68 (64.8)	59 (78.7)	10 (71.4)
Maximum depth: uncertain	9 (4.2)	0 (0.0)	5 (4.8)	4 (5.3)	0 (0.0)

Values are *n* (column %). Body-region categories were not mutually exclusive. TBSA was uncertain for 3 patients, and maximum burn depth was uncertain for 9. This table is descriptive, and no age-group significance tests were performed because several cells were sparse. TBSA, total body surface area.

**Table 2:** Distribution of recorded exposure categories among patients with chemical burns.

Recorded Exposure Category	Patients, *n*	%
Acids, total	64	30.2
Explicitly recorded hydrofluoric acid	23	10.8
Nitric acid	5	2.4
Sulfuric acid	17	8.0
Hydrochloric acid	1	0.5
Acetic acid	3	1.4
Boric acid	1	0.5
Trifluoroacetic acid	3	1.4
Other/acidic mixture	11	5.2
Alkalis, total	63	29.7
Sodium hydroxide	23	10.8
Calcined lime	8	3.8
Other/alkali mixture	32	15.1
Tar or bitumen	9	4.2
Cement	5	2.4
Pesticide	5	2.4
Garlic	4	1.9
Herbal preparation	9	4.2
Antifreeze	5	2.4
Other or insufficiently specified exposure	48	22.6

Percentages are based on *N* = 212. Acid and alkali subcategories are components of their parent totals and should not be summed with the corresponding parent categories. Categories were derived from recorded exposure descriptions and represent database-recorded exposures rather than uniform mechanistic chemical classes. Ambiguous descriptions were retained within the recorded parent group when available; otherwise, they were classified as other or insufficiently specified. “Other/acidic mixture” includes two fluorinated-acid descriptions whose identity could not be resolved; neither was assumed to be hydrofluoric acid.

**Table 3:** Recorded depth and III/IV-degree area among patients with ≤2% TBSA (*N* = 109).

Recorded Maximum-Depth Category	Positive III/IV-Degree Area	Area Recorded as 0%	Area Uncertain	Total
With a full-thickness component	81	4	2	87
No full-thickness component recorded	0	18	0	18
Depth uncertain	0	0	4	4
Total	81	22	6	109

Values are patient counts. Depth was known for 105 patients, and III/IV-degree area for 103. Zero and uncertain area categories were kept separate.

**Table 4:** Recorded care disposition according to demographic and clinical characteristics.

Characteristic	Valid *n*	Healed/No Further Wound Care Recorded, *n* (%)	Outpatient Wound Care, *n* (%)	Readmission, *n* (%)	Critical-Condition Discharge, *n* (%)	*p* Value
Age group, y						0.012
≤17	17	4 (23.5)	13 (76.5)	0 (0.0)	0 (0.0)	
18–40	104	52 (50.0)	42 (40.4)	10 (9.6)	0 (0.0)	
41–60	75	41 (54.7)	33 (44.0)	1 (1.3)	0 (0.0)	
≥61	14	7 (50.0)	6 (42.9)	0 (0.0)	1 (7.1)	
Sex						0.275
Male	156	81 (51.9)	67 (42.9)	8 (5.1)	0 (0.0)	
Female	54	23 (42.6)	27 (50.0)	3 (5.6)	1 (1.9)	
Total body surface area, %						0.006
≤2	109	48 (44.0)	57 (52.3)	4 (3.7)	0 (0.0)	
>2–5	44	24 (54.5)	19 (43.2)	1 (2.3)	0 (0.0)	
>5–10	31	20 (64.5)	10 (32.3)	1 (3.2)	0 (0.0)	
>10–30	16	8 (50.0)	5 (31.2)	3 (18.8)	0 (0.0)	
>30	7	2 (28.6)	2 (28.6)	2 (28.6)	1 (14.3)	
Recorded full-thickness component						0.574
Yes	151	71 (47.0)	72 (47.7)	7 (4.6)	1 (0.7)	
No	50	29 (58.0)	20 (40.0)	1 (2.0)	0 (0.0)	
Overall	210	104 (49.5)	94 (44.8)	11 (5.2)	1 (0.5)	

Percentages are within the valid disposition denominator for each row. Two patients had missing disposition information. *p* values are from two-sided Fisher–Freeman–Halton exact tests. TBSA was uncertain for 3 patients, and maximum recorded depth was uncertain for 9. Patients with uncertain TBSA or maximum depth were excluded from the corresponding comparisons. Disposition was an administrative database field and not a standardized long-term outcome. Recorded full-thickness component denotes a maximum-depth category involving a full-thickness component.

**Table 5:** Hospital length of stay and cost according to demographic and clinical characteristics.

Characteristic	*N*	Length of Stay, d Median (Q1–Q3)	*p* Value	Hospital Cost, RMB Median (Q1–Q3)	*p* Value
Age group, y			0.420		0.508
≤17	18	19.5 (8.2–24.8)		15,574 (6961–25,558)	
18–40	105	13.0 (8.0–29.0)		14,101 (7300–30,035)	
41–60	75	12.0 (6.5–30.0)		12,203 (5972–27,524)	
≥61	14	12.0 (7.2–15.5)		9070 (6305–18,149)	
Sex			0.608		0.155
Male	158	12.5 (8.0–30.0)		13,796 (6921–34,321)	
Female	54	12.0 (7.0–23.8)		12,822 (6236–19,378)	
Total body surface area, %			<0.001		<0.001
≤2	109	12.0 (6.0–22.0)		10,002 (6009–20,305)	
>2–5	44	10.0 (6.8–22.2)		11,345 (5580–20,635)	
>5–10	33	12.0 (8.0–30.0)		16,877 (8948–38,748)	
>10–30	16	23.0 (16.2–59.5)		72,511 (20,089–205,280)	
>30	7	79.0 (40.0–112.5)		665,329 (270,032–1189,441)	
Recorded full-thickness component			0.013		0.016
Yes	152	15.5 (7.0–31.0)		15,305 (6842–36,698)	
No	51	10.0 (7.5–15.0)		10,537 (6024–15,752)	
Overall	212	12.0 (7.0–28.0)		13,488 (6394–28,127)	

Values are medians (Q1–Q3), where Q1 and Q3 denote the first and third quartiles, respectively. RMB indicates Chinese yuan. *p* values are from Kruskal–Wallis tests for age and TBSA groups and Wilcoxon’s rank-sum tests for sex and recorded full-thickness component. Hodges–Lehmann location-shift estimates and 95% confidence intervals for comparisons by recorded full-thickness component are reported in Section 3. TBSA was uncertain for 3 patients, and maximum recorded depth was uncertain for 9. Patients with uncertain TBSA or maximum depth were excluded from the corresponding comparisons. Costs are nominal and were not adjusted for inflation. Analyses are unadjusted and exploratory. Recorded full-thickness component denotes a maximum-depth category involving a full-thickness component.

## Data Availability

The patient-level data are not publicly available because they contain sensitive clinical information and public sharing was not authorized under the study’s ethics approval. Aggregate data supporting the findings of this study are provided in the Supplementary Materials, File S1. Requests for access to de-identified data may be directed to the corresponding author and will be considered in accordance with applicable institutional and ethical requirements.

## Supplementary Materials

The following supporting information can be downloaded at: https://www.mdpi.com/article/10.3390/ebj7030049/s1, Figure S1. Recorded antibacterial-agent selections among patients with chemical burns: (A) Number of recorded antibacterial agents per patient (*N* = 212). (B) Recorded antibacterial-agent selections (*n* = 233); patients could contribute to more than one selection. “No agent recorded” indicates that no antibacterial agent was documented and does not confirm nonuse. “Other agents” comprises the remaining recorded agents not displayed individually. File S1: Aggregate figure data, cohort flow, and coding notes; Table S1: Cross-tabulation of total body surface area (TBSA), source maximum-depth category, and recorded III/IV-degree area; Table S2: Adjusted resource-use models across prespecified model specifications; Table S3: Diagnostics for adjusted resource-use models; Table S4: Sensitivity analyses of the two focal ≤2% TBSA proportions.
